# Alzheimer’s Garden: Understanding Social Behaviors of Patients with Dementia to Improve Their Quality of Life

**DOI:** 10.1007/978-3-030-58805-2_46

**Published:** 2020-08-12

**Authors:** Gloria Bellini, Marco Cipriano, Nicola De Angeli, Jacopo Pio Gargano, Matteo Gianella, Gianluca Goi, Gabriele Rossi, Andrea Masciadri, Sara Comai

**Affiliations:** 8 Institute Integriert Studieren, JKU Linz, Linz, Austria; 9 Jack Baskin School of Engineering, UC Santa Cruz, Santa Cruz, CA USA; 10 Dipartimento di Meccanica, Politecnico di Milano, Milan, Italy; 11 Support Centre for Students with Special Needs, Masaryk University Brno, Brno, Czech Republic; 12 Alta Scuola Politecnica (Politecnico di Milano and Politecnico di Torino), Milano, Italy; 13 Dipartimento di Elettronica, Informazione e Bioingegneria, Politecnico di Milano, Via Ponzio 34/5, 20133 Milano, Italy

**Keywords:** Ambient Assisted Living, Data-driven design, Social behavior, Social wellness assessment

## Abstract

This paper aims at understanding the social behavior of people with dementia through the use of technology, specifically by analyzing localization data of patients of an Alzheimer’s assisted care home in Italy. The analysis will allow to promote social relations by enhancing the facility’s spaces and activities, with the ultimate objective of improving residents’ quality of life. To assess social wellness and evaluate the effectiveness of the village areas and activities, this work introduces measures of sociability for both residents and places. Our data analysis is based on classical statistical methods and innovative machine learning techniques. First, we analyze the correlation between relational indicators and factors such as the outdoor temperature and the patients’ movements inside the facility. Then, we use statistical and accessibility analyses to determine the spaces residents appreciate the most and those in need of enhancements. We observe that patients’ sociability is strongly related to the considered factors. From our analysis, outdoor areas result less frequented and need spatial redesign to promote accessibility and attendance among patients. The data awareness obtained from our analysis will also be of great help to caregivers, doctors, and psychologists to enhance assisted care home social activities, adjust patient-specific treatments, and deepen the comprehension of the disease.

## Introduction

In 1990, only 6% of the world population was aged 65 years or over, 10% in 2019 and by 2050 the number is projected to rise to 15%, potentially surpassing that of adolescents, according to the United Nations forecast [1]. As a consequence, requests for assistance, which are already on the rise and having an impact on health-care systems worldwide, will further intensify. One of the leading causes of dependency and disability in the elderly is dementia, a chronic degenerative disease that affects memory, visuospatial abilities, domain and functional cognition, attention, and problem solving capabilities. The number of affected people is quite high: 5–8% of people aged 60 and over suffer from dementia, rising to 50% when considering people over 85 [2].

Alzheimer’s disease (AD) is the most frequent form of dementia consisting in a severe progressive neurological pathology in which the main cognitive functions of an individual are compromised. This disease significantly affects the quality of life and, so far, medical sciences have not been able to find an effective treatment to halt or reverse its progression. However, recent studies have found that engaging in social relationships and activities could delay the cognitive decline of AD patients [3].

Alzheimer’s Garden is a joint project of Politecnico di Milano and Politecnico di Torino that aims to understand the social behavior of AD patients living in a healthcare facility and to promote sociability among them to improve their quality of life and slow down the progression of the disease. The subject of our case study is *Il Paese Ritrovato* (see Sect. 3), an Ambient Assisted Living (AAL) facility in Italy featuring an advanced technological infrastructure. Specifically, this project assesses both the degree of relations among residents and the popularity of the facility spaces as an indicator of accessibility by measuring the attendance of the patients throughout an observation period, identifying the most preferred locations and the ones in need of enhancements.

## State of the Art

Activities play a crucial role in enabling people with AD to live a life as satisfying as possible, allowing them to pursue their own hobbies and interests, creating immediate pleasure, restoring dignity and enabling friendships [4]. It has been shown that high social engagement reduces the rate of cognitive decline by 91% [5]. On the contrary, both actual social isolation, including having a small social network and participating in few activities with others, and perceived social isolation, that is, feeling lonely, are robustly associated with AD progression and cognitive decline [6].

Spatial solutions, especially those focused on accessibility, play an important role in promoting socialization between patients. Van Hecke et al. [7] state that when AD patients are not allowed to leave a dementia special care unit, it is important to provide sufficient freedom of movement and social interaction within the unit, including access to private outdoor space. This freedom of movement is crucial for patients walking through the unit without a specific destination. This statement is reinforced by the research of Ferdous et al. [8] proving architectural configuration affects the type of conversations likely to occur in certain locations within assisted care homes and, consequently, social relations.

The research team Lab.I.R.Int of the Design Department of Politecnico di Milano with Lapo Lani and Ivo Cilesi describes the concept of *therapeutic habitat* to define good design for Alzheimer’s patients [9]. It recognizes the value of intangible environmental features that act as activators of opportunities for social relationships, conversations and daily rituals, improving the wellbeing of patients. Several outdoor environments do not adequately address accessibility issues concerning people with AD, as they often are disorientating, difficult to interpret and navigate, and threatening or distressing for them [10].

## Case Study: Il Paese Ritrovato

*Il Paese Ritrovato* [11] is the first Alzheimer’s assisted care home in the form of a village ever built in Italy. Officially opened in 2018 by La Meridiana [12] in the city of Monza, the village is developed as a traditional, small Italian town with streets, squares and gardens. The facility is composed of 8 units, each hosting 8 single rooms with private services and common areas, accounting for a total of 64 residents. At its core there are common facilities such as a church, a café, shops and a theatre to recreate the safe and stimulating environment typical of a small village. Several brain-stimulating activities are organized in multisensory rooms and labs, including pet therapy, music therapy, aromatherapy, and entertainment clubs. Building exteriors are painted each with a different color, taking inspiration from those of the close neighborhoods of Monza to evoke a feeling of resemblance in residents as if they were walking in familiar streets. Caregivers assume different roles beside their own (e.g., gardener, hairdresser, barista) to make the whole environment even more realistic.

The typical resident of *Il Paese Ritrovato* is a person with a fair level of self-reliance and independence, able to move around the village autonomously. All residents were diagnosed with mild to moderate AD, in the worst cases resulting in spontaneous confabulation, temporal and spatial context confusion, and personality and behavioral changes leading to occasional delirium [13]. Residents usually establish strong bonds with caregivers, who play a fundamental role in their daily routines, including eating together and conducting social activities.

## Methodology and Results

A monitoring system, previously implemented by Masciadri et al. [14], collects residents’ localization data through a Received Signal Strength Indicator (RSSI) based smart wearable bracelet every 10 seconds and stores the history of visited areas inside the village for every resident into a database at the end of each day [14]. Residents are informed about the localization system, and gave the rights to La Meridiana Due who is responsible for the data collection and treatment process. The necessary data for this study was preprocessed to guarantee the anonymity of the residents before being provided to the research team.

Taking into account the data at our disposal and following our main objectives, we develop our analysis on two main aspects: residents’ social interactions and their behavior towards the village areas.

### Assessing Patients Sociability: The Relational Index

**Fig. 1.**
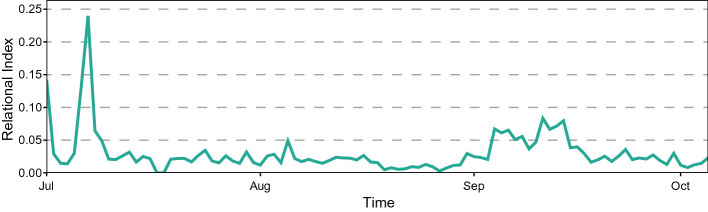
An example of patient’s Individual Relational Index plot

**Fig. 2.**
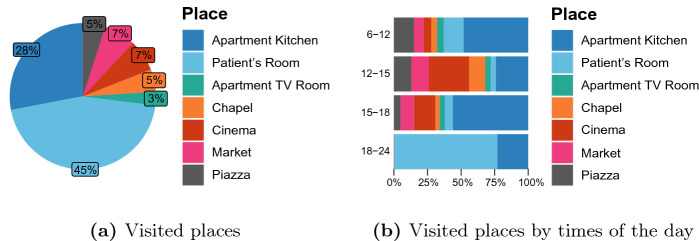
An example of patient’s Localization Profile

Assessing residents’ wellbeing, specifically their social behavior, only through their localization data is a hard task. We resort to the Relational Index $RI_{i,j}^{d}$ [14]: an indicator that aims to estimate the degree of relation between persons *i* and *j* on day *d* taking into account the amount of time they spend together in the same place. We extend it by introducing the Individual Relational Index for person *i* on day *d*:

1 $$\begin{aligned} RI_{i}^d=\frac{\sum _{j\in A^d\setminus \{i\}}{RI_{i,j}^{d}}^2}{|A^d|-1}, \end{aligned}$$

where $A^d$ is the set of active users on day *d*. This index estimates quite accurately the sociability of person *i* on day *d*, and can be further extended to the entire community, for instance, by averaging over its members. Observing the value of the Relational Index and its trend over time, it is possible to discriminate between more and less sociable patients outlining a social profile for each of them. Furthermore, social isolation can be easily identified by observing decays in the patient’s Individual Relational Index curve and changes in the visited places over time. Figure 1 shows an example plot of the Individual Relational Index. The change in late September could be investigated further by doctors and caregivers. Figure 2 shows an example of user profiling based on the Relational Index for a quite sociable resident. Figure 2a shows the average percentage of time spent in the village areas during a period of time of fifteen days, while Fig. 2b specifies it over the different times of the day.

In our analysis, we perform a *multiple linear regression* in which the Relational Index is the response variable. Applying *ordinary least squares* we estimate the correlation between the Relational Index and the following variables: season, temperature, weather, patient’s bedroom floor (ground or first), predisposition to walk on a specific day, and patient’s average hourly walked distance. Furthermore, we build a *contingency matrix* and perform a $\chi ^2$ *independence test* to study the correlation of the Relational Index with the aforementioned variables considered individually.

These two models were chosen because of their high reliability, their statistical relevance, and consistency. The low *p-values* obtained from the linear regression and the dependencies in the contingency table highlight the strong correlation between the considered variables and the patients’ sociability. The only irrelevant variable is the weather. This is both due to its multicollinearity with other variables such as temperature and season, but also to the fact that most facilities are indoors, making this factor less influential on the behavior of residents. Our analysis confirms that accommodating residents with dementia on the top floor should only occur when strictly necessary (e.g., to prevent patients exiting from dementia special care units), adopting thoughtfully designed solutions, as Van Hecke et al. state in [7].

### Assessing Places Sociability: The Popularity Index

**Fig. 3.**
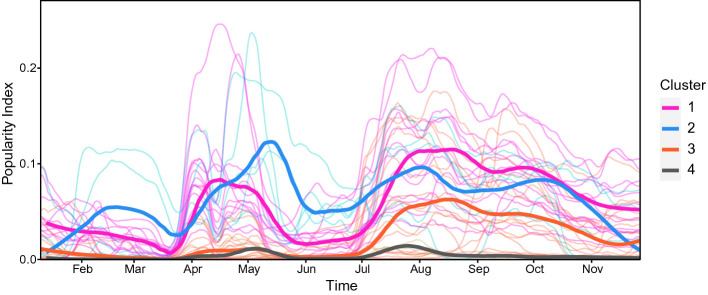
Popularity Index plot and identified clusters of places

We analyze the way places are visited by residents identifying highly frequented areas and unpopular ones. We introduce the Popularity Index: a measure of the attendance of a place. Namely, let $n_{p}^{d}(h)$ be the number of detected individuals in place *p* over an hour starting from hour *h* on day *d*. Then, the Popularity Index of place *p* on day *d* is defined as:

2 $$\begin{aligned} PI_{p}^{d} = \frac{1}{|A^{d}||D|}\sum _{h\in D}n_{p}^{d}(h), \end{aligned}$$

where $A^d$ is the set of active users on day *d*, and *D* is the set of hours in which users are generally awake. We apply typical methodologies proper of Functional Data Analysis [15] in order to detect common features and patterns among the places of the facility. We first embed data in a suitable space through a smoothing procedure. Then, we perform *functional k-means clustering* [16] detecting non-trivial and peculiar trends exhibiting some sort of time dependence (see Fig. 3), thus obtaining four clusters of places.

**Fig. 4.**
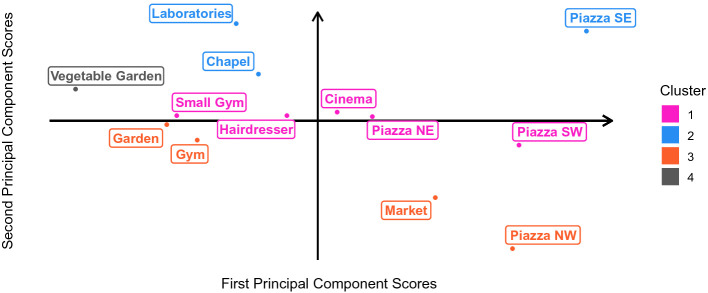
Functional components scores scatter plot for public social places. Places on the right (left) are quite popular (unpopular) among residents; those at the top (bottom) are more popular during winter and spring (summer and autumn). Places on the left definitely need enhancements.

The thicker curves in the foreground, representing the identified clusters, are the main Popularity Index trends distinguished inside *Il Paese Ritrovato*: we observe which places are more frequented (clusters 1 and 2) and which are less popular (clusters 3 and 4). Places belonging to the different clusters are depicted in Fig. 4. Note that cluster 2 is generally more popular than cluster 1 during the first half of the year, while we may observe an opposite behavior in the second half; cluster 3 shows an increasing popularity in the second half of the observance period; cluster 4 is stable and represents those areas that are barely visited by the residents.

This procedure groups together places exhibiting a similar trend during the observation period. As a consequence, the metric introduced for clustering is blind towards constant shifts. More specifically, clusters 1 and 2 show higher curves, but only represent an average trend. Hence, places belonging to these clusters may be not so popular.

To enrich the aforementioned results, we perform a *functional principal component analysis* (FPCA) [17] detecting those features clustering may leave unseen. Through the first two principal components, obtained as perturbations with respect to the average curve, we are able to explain more than 80% of the total variance. The considered principal components are summarized through FPCA scores, preserving the respective component interpretation and resulting in a clearer graphical representation. The score of the first principal component captures the variance in the popularity of a place with respect to the average value for the whole observation period. Considering the two halves of the observation period, the score of the second principal component rewards a place for its popularity in the first half and penalizes it for its popularity in the second half.

This analysis truly extends the results obtained through clustering, since we can observe strong coherence in the outcomes, constituting highly reliable and valid hypotheses for future interventions. Combining the results of the presented analyses, we can clearly identify neglected places in need of enhancements. For instance, the outdoor areas, namely the vegetable garden and the garden, are rarely frequented.

### Accessibility and Neglected Spaces

*Il Paese Ritrovato* is a modern ensemble of buildings and does not present accessibility issues attributable to architectural barriers. The main spaces for community life in the village are located on the ground floor and are arranged in the center of the square to be easily identifiable and available to the residents. The two main outdoor locations of the village, the garden and the vegetable garden, are also easily accessible. However, these spaces cover peripheral areas of the village, being situated on the edges of the structure. This causes the residents to rarely spend time outside, as they usually do not pass by these open spaces unless it is their intention.

It would be desirable to make these areas more attractive, as gardens provide excellent sensory stimuli to the residents. Olfactory and auditory stimuli are extremely important for their mental health, since the associations between senses and memories are hardly affected by AD progression [18]. We therefore aim to work on intangible features of the external environment such as sounds, smells, lights, and climate to create therapeutic micro-habitats that provide tactile and sound stimuli, therefore improving residents wellbeing.

## Conclusions and Future Work

In this work, we analyzed localization data to gather insights on the behavior of the residents of *Il Paese Ritrovato* to design solutions for the improvement of their social life and wellbeing. We extended the notion of Relational Index [14] to single individuals and investigated its statistical correlation with other variables by means of a *linear regression* and a $\chi ^2$ *independence test*, observing and explaining why it is influenced by all of them except for the weather. We also examined the popularity of different areas of the facility by leveraging *functional k-means clustering* and *functional principal component analysis*, identifying well-functioning social areas and those in need of enhancements, such as the outdoor places.

The insight obtained from the analysis performed on the Relational Index could be game-changing for caregivers and psychologists, contributing to the overall assessment of patients wellbeing. Monitoring the Relational Index trend identifying decays - indicating the patient is starting to isolate themselves - could also offer a precious hint to doctors on the disease progression, leading to the development of better personalized treatments. Moreover, comprehending the way both domain specific and domain agnostic variables influence socialization could be used to build advanced predictive tools for the estimation of the Relational Index over time. This measure can be also used to study the relationship between two specific patients, spotting recurring behavioral patterns and identifying best friendships.

Furthermore, we intend to combine the statistical and accessibility analyses of the social places of *Il Paese Ritrovato* to plan, propose and finally introduce new social activities, as well as spatial modifications to the village facilities, such as sensory maps. Once the activity revision and space redesign are carried out, we plan to evaluate them through further detailed analyses. For instance, as a graphical tool, we intend to design the social network of the village where the connection between two residents depends on their Relational Index. This will serve to observe the formation of social clusters and analyze their change over time.

Finally, we plan to implement a dynamic social activities scheduler aimed to promote active aging through the daily proposal of new engaging activities, including physical ones to exploit their positive effects [19], tailored to the preferences of residents, as described by Costa et al. [20], in a user-centered perspective.
